# Comparison of Second-Line Quadruple Therapies with or without Bismuth for *Helicobacter pylori* Infection

**DOI:** 10.1155/2015/163960

**Published:** 2015-05-18

**Authors:** Guang-Hong Jheng, I-Chen Wu, Hsiang-Yao Shih, Meng-Chieh Wu, Fu-Chen Kuo, Huang-Ming Hu, Chung-Jung Liu, Wen-Hung Hsu, Chi-Tan Hu, Ming-Jong Bair, Chao-Hung Kuo, Deng-Chyang Wu, Ping-I Hsu

**Affiliations:** ^1^Graduate Institute of Clinical Medicine, Kaohsiung Medical University, Kaohsiung City 807, Taiwan; ^2^Department of Internal Medicine, Kaohsiung Municipal United Hospital, Kaohsiung City 804, Taiwan; ^3^Division of Gastroenterology, Department of Internal Medicine, Kaohsiung Medical University Hospital, Kaohsiung 807, Taiwan; ^4^Department of Medicine, Faculty of Medicine, College of Medicine, Kaohsiung Medical University, Kaohsiung City 807, Taiwan; ^5^Center for Stem Cell Research, Kaohsiung Medical University, Kaohsiung City 807, Taiwan; ^6^Department of Internal Medicine, Kaohsiung Municipal Hsiao-Kang Hospital, Kaohsiung City 812, Taiwan; ^7^School of Medicine, College of Medicine, E-Da Hospital, I-Shou University, Kaohsiung City 824, Taiwan; ^8^Division of Gastroenterology, Department of Internal Medicine, Buddhist Tzu Chi General Hospital and School of Medicine, Tzu Chi University, Hualien 970, Taiwan; ^9^Division of Gastroenterology, Department of Internal Medicine, Mackay Memorial Hospital, Taitung Branch, Taitung City 950, Taiwan; ^10^Department of Internal Medicine, Kaohsiung Municipal Cijin Hospital, Kaohsiung City 812, Taiwan; ^11^Center for Infectious Disease and Cancer Research, Kaohsiung Medical University, Kaohsiung City 807, Taiwan; ^12^Division of Gastroenterology, Department of Internal Medicine, Kaohsiung Veterans General Hospital, National Yang-Ming University, Kaohsiung 813, Taiwan

## Abstract

The bismuth-based quadruple regimen has been applied in *Helicobacter pylori* rescue therapy worldwide. The non-bismuth-based quadruple therapy or “concomitant therapy” is an alternative option in first-line eradication but has not been used in second-line therapy. Discovering a valid regimen for rescue therapy in bismuth-unavailable countries is important. We conducted a randomized controlled trial to compare the efficacies of the standard quadruple therapy and a modified concomitant regimen. One hundred and twenty-four patients were randomly assigned into two groups: RBTM (rabeprozole 20 mg bid., bismuth subcitrate 120 mg qid, tetracycline 500 mg qid, and metronidazole 250 mg qid) and RATM (rabeprozole 20 mg bid., amoxicillin 1 g bid., tetracycline 500 mg qid, and metronidazole 250 mg qid) for 10 days. The eradication rate of the RBTM and RATM regimen was 92.1% and 90.2%, respectively, in intention-to-treat analysis. Patients in both groups had good compliance (~96%). The overall incidence of adverse events was higher in the RATM group (42.6% versus 22.2%, *P* = 0.02), but only seven patients (11.5%) experienced grades 2-3 events. In conclusion, both regimens had good efficacy, compliance, and acceptable side effects. The 10-day RATM treatment could be an alternative rescue therapy in bismuth-unavailable countries.

## 1. Introduction

*Helicobacter pylori (H. pylori)* causes several gastrointestinal diseases including peptic ulcers, gastric adenocarcinoma, and mucosa associated lymphoid tissue lymphoma (MALToma); eradication of* H. pylori* is recommended in these conditions [1]. The standard 7-day triple therapy including a proton pump inhibitor (PPI), amoxicillin, and clarithromycin is the first-line treatment for* H. pylori*. However, its failure rate has increased to almost 20% in Taiwan [2, 3] and around 60% of countries worldwide fail to reach an eradication rate of more than 80% [4–6]. The standard quadruple therapy consisting of PPI, bismuth salt, tetracycline, and metronidazole is widely used as the first-line treatment if clarithromycin resistance rate is more than 20%. The 3rd Brazilian consensus, 2013, and Maastricht IV consensus [7, 8] also recommended it as a second-line salvage therapy. However, bismuth is not available in many countries; thus, an equally effective non-bismuth-based quadruple therapy is essential for* H. pylori* treatment [9].

The non-bismuth-based quadruple therapy, consisting of the standard triple therapy (PPI, amoxicillin, and clarithromycin) plus either metronidazole or tinidazole, is also known as “concomitant therapy” [9]. It has been used as an alternative first-line eradication regimen [10, 11]. However, clarithromycin has been included in the first-line triple therapy and the secondary* H. pylori* resistance rates in Taiwan are higher in clarithromycin (29.7–45.7%) and metronidazole (40–58.7%) and lower in amoxicillin (4.3~6%) and tetracycline (0%) [3, 12, 13]. Therefore, we modified the standard concomitant therapy by omitting clarithromycin and designed a randomized study to compare the performance of two rescue regimens: RBTM (rabeprazole, bismuth subcitrate, tetracycline, and metronidazole) and RATM (rabeprazole, amoxicillin, tetracycline, and metronidazole). To the best of our knowledge, it is the first study to directly compare the two regimens as the second-line therapy.

## 2. Material and Methods

### 2.1. Study Population, Therapy Protocols, and Confirmation of* H. pylori* Status

All patients who had persistent* H. pylori* infection after the standard first-line triple therapy (PPI bid., clarithromycin 500 mg bid., and amoxicillin 1 g bid. for 7 days) were enrolled from two medical centers, Kaohsiung Medical University Hospital and Kaohsiung Veterans General Hospital in Kaohsiung, Taiwan, between November 2009 and October 2011. The rapid urease test, histology, and culture were not performed in all patients. Some patients only received ^13^C urea breath test to confirm the presence of* H. pylori*. Hence the definition of “the presence of* H. pylori*” was (1) positive results of both rapid urease test and histology, (2) positive culture result, or (3) positive finding of ^13^C urea breath test. The exam of rapid urease test, histology, and culture was performed in 79 patients. The results of culture revealed 35 positive findings and 44 negative findings. In the 44 patients with negative finding of* H. pylori* culture was confirmed by positive results of both rapid urease test and histology. The rest of patients in this study only received ^13^C urea breath test to confirm the presence of* H. pylori*. The exclusion criteria included (a) ingestion of antibiotics, bismuth, or PPI within 4 weeks before our intervention; (b) a history of allergy to the medications used; (c) previous gastric surgery; (d) the coexistence of serious concomitant illness such as decompensated liver cirrhosis and uremia; and (e) pregnant or lactating women.

The participants were randomly assigned into the 10-day treatment groups by using a computer number table. The RBTM regimen consisted of rabeprazole 20 mg bid, bismuth subcitrate 120 mg qid, tetracycline 500 mg qid, and metronidazole 250 mg qid, and the RATM consisted of rabeprazole 20 mg bid, amoxicillin 1 g bid, tetracycline 500 mg qid, and metronidazole 250 mg qid. The participants were asked to return 1-2 weeks after the treatment course for a questionnaire interview and to count the residual tablets. ^13^C urea breath test was performed to confirm their* H. pylori* status 4 weeks later. All participants gave written informed consent. This study was approved by the Institutional Review Board of Kaohsiung Medical University.

### 2.2. Questionnaire

The indexes of questions included sex, age, underlying systemic disease, and smoking and alcohol-drinking habits. The details of adverse effects in the questionnaire included diarrhea, constipation, abdominal pain, anorexia, nausea, vomiting, skin rash, headache, dizziness, bad taste, and fatigue, among others. We differentiated the different degrees of adverse effect into four grades including 0: none; 1: feeling discomfort but can take daily activity and work normally; 2: feeling discomfort and affecting their daily activity or work; 3: feeling too much discomfort to take the drug, causing discontinuation of the treatment course. The definition of poor compliance was completing the therapy course of less than 70% [14].

### 2.3. Statistical Analysis

The* H. pylori* eradication rates were evaluated by intention-to-treat (ITT) and per-protocol (PP) analyses. ITT analysis was defined as comparing all patients enrolled in the two groups. Those who did not return for a ^13^C urea breath test were deemed as dropout. PP analysis was defined as comparing two groups of patients who completed the whole treatment course and received* H. pylori* follow-up. The characteristics, eradication rates, and presence of adverse events were calculatedly by the Chi-square test. Student's *t*-test was used to compare the patient's ages in the two groups. A *P* value less than 0.05 was considered statistically significant and all *P* values were two-sided. The software of SPSS was used for statistical analysis (IBM Corp. version 19). Assuming that the eradication rate of the RBTM group was 70% [3], and the RATM group achieved a 90% eradication rate [15], a 20% increase, our statistical power in this study is 80% under the sample size of about 60 subjects in each group and the two-sided *P* value is 0.05 if 95% of patients completed the follow-up.

## 3. Results

**Figure 1:**
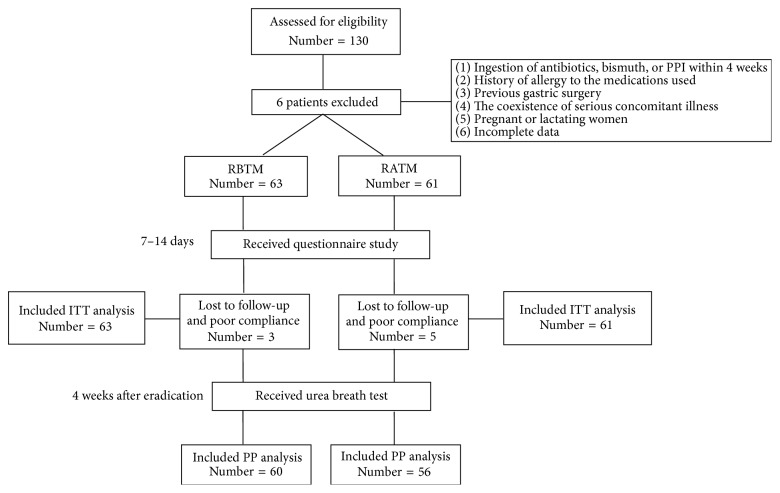
Flow diaphragm of study design through randomization.

The flow chart of study design and randomization protocol is shown in Figure 1. One hundred and thirty patients were enrolled in this study; six of them were excluded according to exclusion criteria. The remaining 124 patients were randomly assigned into the RBTM (*N* = 63) and RATM (*N* = 61) groups. One patient in the RBTM group and three patients in the RATM group did not return to confirm* H. pylori* status and were deemed dropout in the ITT analysis. Two patients in the RBTM group and two patients in the RATM group completed less than 70% of therapy course and were deemed incomplete therapy course in the ITT analysis.

The demographic characteristics of study participants were not significantly different between the two groups. The most common endoscopic diagnosis in our study was gastritis (RBTM: 46.0% versus RATM: 42.6%), followed by duodenal ulcer (RBTM: 27.0% versus RATM: 37.7%) (Table 1). The* H. pylori* eradication rates of the RBTM and RATM regimens were 92.1% versus 90.2% in ITT analysis and 93.3% versus 89.3% in PP analysis. The compliance between the two groups was also similar (RBTM: 96.8% versus RATM: 96.7%, *P* = 0.97). The overall rate of adverse events was 22.2% (14/63) in the RBTM group and 42.6% (26/61) in the RATM group (*P* = 0.02) (Table 2). Although more adverse events were reported in the RATM group, only seven patients had severity more than grades 2 or 3. The rest of the study participants with discomfort experience were only assessed at grade 1. Dizziness (8 versus 0 cases, *P* = 0.03) and headache (7 versus 1 case, *P* = 0.08) were more common in the RATM than in the RBTM group. The most common adverse event in the RATM group was nausea (*N* = 12) (Table 3).

## 4. Discussion

The Maastricht IV consensus has suggested that metronidazole should be included in the standard second-line quadruple therapy [7]. The concomitant or non-bismuth-based quadruple therapy has not been used as the second-line treatment yet. By replacing clarithromycin with tetracycline, we directly compared the modified concomitant regimen (RATM) with the bismuth-based quadruple therapy (RBTM) for 10 days. We found a comparable efficacy (RBTM 92.1% versus RATM 90.2% in ITT analysis) and compliance (~96%), but variable adverse effects (RBTM 22.2% versus RATM 42.6%, *P* = 0.02) of the rescue quadruple therapies with or without bismuth. The eradication rates of both 10-day regimens were similar to the 14-day quadruple therapy (ITT: 82.6%, PP: 93.6%) [16]. The costs of both regimens were much cheaper than levofloxacin-containing rescue therapy. Moreover, RATM can be a useful alternative in bismuth-unavailable areas. All participants received the same first-line treatment in the study hospitals. Thus, we had a more homogeneous study population.

Only one study used the same antibiotic combination and compared the concomitant therapy (esomeprazole, amoxicillin, tetracycline, and metronidazole) with bismuth-based quadruple therapy (esomeprazole, bismuth, tetracycline, and metronidazole) in the first-line treatment [17]. The eradication rates were ITT: 74% versus 79% and PP: 80.4% versus 89.7%. Moreover, a meta-analysis of the first-line concomitant therapy consisting of PPI, amoxicillin, and clarithromycin plus either metronidazole or tinidazole revealed an 88% eradication rate in ITT analysis [15]. Many studies have compared different bismuth-based quadruple therapies containing different proton pump inhibitors or antibiotics for a variable treatment duration as the second-line treatment. The recently reported eradication rates were 63.9–85.1% in ITT analysis and 82.6–96.2% in PP analysis [3, 12, 14, 16, 18, 19]. The RBTM regimen in our study seems to have a better result.

*H. pylori* eradication is influenced by many factors, such as antibiotic resistance, therapy duration, drug compliance, intragastric acidity, and CYP2C19 genetic polymorphism [20]. Regarding antibiotic resistance, the worldwide primary* H. pylori*-resistant rates to clarithromycin, metronidazole, amoxicillin, and tetracycline were 17.2%, 26.7%, 11.2%, and 5.9%, respectively [21]. More specifically, in Asia, the clarithromycin (18.9%) and metronidazole (37.1%) resistance is higher, while tetracycline resistance (2.4%) is lower than average [21]. Our previous studies found that the primary resistant rates were 6.6~13.2% to clarithromycin, 26.7~56% to metronidazole, 0~2% to amoxicillin, and 0.6% to tetracycline [2, 22–24]. Moreover, secondary* H. pylori* resistance was even higher to clarithromycin (29.7~45.7%) and metronidazole (40~58.7%), while being similar to amoxicillin (4.3~6%) and tetracycline (0%) [3, 12, 13]. Dual clarithromycin and metronidazole resistance is an important factor influencing the eradication efficacy. Chi et al. reported a 16–18% dual resistance rate in Taiwan and suggested that second-line quadruple therapy including tetracycline and amoxicillin could improve the eradication efficacy [13]. However, our previous study found that the efficacy of concomitant therapy was not affected by dual resistance (75.0% versus 92.4%, *P* = 0.22) [23]. Despite the high metronidazole resistance in many areas, Katelaris et al. proposed that metronidazole resistance would not be a major cause of quadruple therapy failure because adding PPI with bismuth triple therapy would overcome the high resistance rate of metronidazole [25]. Kuo et al. indicated that longer metronidazole usage (at least 7 days) in second-line therapy could conquer the metronidazole resistant rate and reach a desirable result (ITT: 79%, PP: 91%) [12]. The suggested dose of metronidazole in rescue quadruple therapy varies from 1000 to 2000 mg daily. We chose 1000 mg daily according to the satisfying efficacy of previous studies and better drug compliance [3, 12, 14, 25]. A Korean trial and the review article have suggested that an extended duration up to 10–14 days was more adequate in rescue therapy [16, 26]. In this study, we used metronidazole 250 mg qid for 10 days and found a good result. One of the limitations of this study is lack of information on antibiotic resistance.

We chose rabeprazole-based regimens in this study to minimize the effect of* CYP2C19* polymorphism on PPI clearance and intragastric acidity [27, 28]. The* CYP2C19* polymorphism leads to three phenotypes: the homozygous extensive metabolizer, heterozygous extensive metabolizer, and poor metabolizer. The poor metabolizer is associated with superior efficacy in curing* H. pylori* because of slower PPI clearance and higher intragastric pH level and, thus, higher intragastric concentrations of antibiotics [28]. Although we did not check CYP2C19 polymorphism in this study, 20% of Asian people have poor metabolizing genotype, which is higher than in Western populations [27]. Therefore, the influence of CYP2C19 polymorphism is not considered high here.

The overall adverse effect was more common in the RATM than in the RBTM group (42.6% versus 22.2%). However, the incidence of grades 2-3 events in our RATM group was only 11.5% (7 patients). The only study using amoxicillin, tetracycline, and metronidazole-containing regimens reported an overall adverse event rate of 10% [17]. The most common adverse event in our RATM group was nausea (*N* = 12), but only four patients had grades 2-3 events. Moreover, there were more adverse events when the treatment course got longer [19, 20]. In our RBTM group, the most common side effect was vomiting (10%, 6 cases), which was similar to other reports [18–20]. Nevertheless, in our previous studies, nausea was the most comment event in bismuth-based quadruple therapy [3, 12, 14]. There was a wide range of adverse event incidences (15–46.4%) using quadruple therapy containing PPI, bismuth, tetracycline, and metronidazole, and metallic taste, nausea, vomiting, and headache were most commonly complained of [3, 12, 14, 18–20]. Our overall adverse event rate (22.2%) in the RBTM group was compatible with other studies. A review and meta-analysis found no serious side effect to bismuth-based* H. pylori* eradication unless the subject had allergy to these drugs [29]. Both RBTM and RATM were safe, well tolerated, and with good compliance in our trial. Only two patients in the RATM group had poor compliance due to skin rash (*n* = 1) and unknown reason (*n* = 1). In the RBTM group, two patients took less than 70% of the medication because of nausea and vomiting. However, three of them (75%) had successful* H. pylori* eradication.

In conclusion, the RATM concomitant therapy as a second-line treatment had similar efficacy but more adverse events than the bismuth-based quadruple therapy. It could be an alternative in bismuth-unavailable areas or where intolerance to bismuth is noted. Further randomized study is needed to investigate the influence of secondary antibiotic resistances on the treatment effects.

## Figures and Tables

**Table 1:** Characteristics of the participants receiving different eradication regimens.

	RBTM group (*n* = 63)	RATM group (*n* = 61)	*P* value
Age (years)	55.0 ± 12.1	54.1 ± 12.0	0.68
Sex			
Male	24 (38.1%)	33 (54.1%)	0.07
Female	39 (61.9%)	28 (45.9%)	
Smoking	5 (7.9%)	7 (11.5%)	0.51
Alcohol drinking	3 (4.8%)	2 (3.3%)	0.68
Diagnosis			
Gastritis	29 (46.0%)	26 (42.6%)	0.57
Gastric ulcer	7 (11.1%)	7 (11.5%)	
Duodenal ulcer	17 (27.0%)	23 (37.7%)	
Peptic ulcer^*^	1 (1.6%)	1 (1.6%)	
Others	9 (14.3%)	4 (6.6%)	

^*^Peptic ulcer: concurrent gastric ulcer and duodenal ulcer.

RBTM: rabeprazole, bismuth subcitrate, tetracycline, and metronidazole.

RATM: rabeprazole, amoxicillin, tetracycline, and metronidazole.

**Table 2:** The outcomes of RBTM and RATM treatment regimens.

	RBTM group (*n* = 63)	RATM group (*n* = 61)	*P* value
Eradication rate			
Intention-to-treat	92.1% (58/63)	90.2% (55/61)	0.71
Per-protocol	93.3% (56/60)	89.3% (50/56)	0.44
Compliance	96.8% (61/63)	96.7% (59/61)	0.97
Adverse events	22.2% (14/63)	42.6% (26/61)	0.02

**Table 3:** Adverse events of the RBTM and RATM regimens.

Adverse events	RBTM (*n* = 63)	RATM (*n* = 61)	*P* value
Diarrhea	2 (2/0/0)	6 (5/1/0)	0.28
Constipation	0 (0/0/0)	0 (0/0/0)	—
Abdominal pain	3 (3/0/0)	7 (5/2/0)	0.25
Anorexia	1 (1/0/0)	2 (0/1/1)	0.39
Nausea	5 (5/0/0)	12 (8/2/2)	0.14
Vomiting	6 (6/0/0)	10 (8/1/1)	0.46
Skin rash	1 (1/0/0)	2 (0/2/0)	0.22
Headache	1 (1/0/0)	7 (6/1/0)	0.08
Dizziness	0 (0/0/0)	8 (3/3/2)	0.03
Bad taste	2 (2/0/0)	4 (2/2/0)	0.35
Fatigue	2 (2/0/0)	5 (4/1/0)	0.40
Others	0 (0/0/0)	4 (3/0/1)	0.12

Total number of individual adverse events (number of different degrees of adverse events: 1/2/3).
